# Different Roles for Tet1 and Tet2 Proteins in Reprogramming-Mediated Erasure of Imprints Induced by EGC Fusion

**DOI:** 10.1016/j.molcel.2013.03.011

**Published:** 2013-03-28

**Authors:** Francesco M. Piccolo, Hakan Bagci, Karen E. Brown, David Landeira, Jorge Soza-Ried, Amelie Feytout, Dylan Mooijman, Petra Hajkova, Harry G. Leitch, Takashi Tada, Skirmantas Kriaucionis, Meelad M. Dawlaty, Rudolf Jaenisch, Matthias Merkenschlager, Amanda G. Fisher

(Molecular Cell *49*, 1023–1033, March 28, 2013)

Takashi Tada’s name was previously misspelled on the version of this paper that published online on February 28, 2013. The correct spelling appears above. The authors apologize for any inconvenience.

